# Rett Syndrome and Fragile X Syndrome: Different Etiology With Common Molecular Dysfunctions

**DOI:** 10.3389/fncel.2021.764761

**Published:** 2021-11-19

**Authors:** Snow Bach, Stephen Shovlin, Michael Moriarty, Barbara Bardoni, Daniela Tropea

**Affiliations:** ^1^School of Mathematical Sciences, Dublin City University, Dublin, Ireland; ^2^Neuropsychiatric Genetics, Department of Psychiatry, School of Medicine, Trinity College Dublin, Trinity Translational Medicine Institute, St James’s Hospital, Dublin, Ireland; ^3^School of Medicine, Trinity College Dublin, Dublin, Ireland; ^4^Inserm, CNRS UMR 7275, Institute of Molecular and Cellular Pharmacology, Université Côte d’Azur, Valbonne, France; ^5^Trinity College Institute of Neuroscience, Trinity College Dublin, Dublin, Ireland; ^6^FutureNeuro, The SFI Research Centre for Chronic and Rare Neurological Diseases, Dublin, Ireland

**Keywords:** Rett syndrome, Fragile X syndrome, synaptic plasticity, FMRP, MeCP2, neurodevelopmental disorders

## Abstract

Rett syndrome (RTT) and Fragile X syndrome (FXS) are two monogenetic neurodevelopmental disorders with complex clinical presentations. RTT is caused by mutations in the Methyl-CpG binding protein 2 gene (*MECP2*) altering the function of its protein product MeCP2. MeCP2 modulates gene expression by binding methylated CpG dinucleotides, and by interacting with transcription factors. FXS is caused by the silencing of the *FMR1* gene encoding the Fragile X Mental Retardation Protein (FMRP), a RNA binding protein involved in multiple steps of RNA metabolism, and modulating the translation of thousands of proteins including a large set of synaptic proteins. Despite differences in genetic etiology, there are overlapping features in RTT and FXS, possibly due to interactions between MeCP2 and FMRP, and to the regulation of pathways resulting in dysregulation of common molecular signaling. Furthermore, basic physiological mechanisms are regulated by these proteins and might concur to the pathophysiology of both syndromes. Considering that RTT and FXS are disorders affecting brain development, and that most of the common targets of MeCP2 and FMRP are involved in brain activity, we discuss the mechanisms of synaptic function and plasticity altered in RTT and FXS, and we consider the similarities and the differences between these two disorders.

## Introduction

Rett syndrome (RTT) and Fragile X syndrome (FXS) are neurodevelopmental disorders associated with mutations in genes located on the X chromosome. Hence, in both RTT and FXS, the presentation is more severe in male patients, and the reduced severity in females is to be attributed to the presence of two copies of the genes, although X-inactivation influences the number of copies that remain active.

Patients with RTT show apparent normal development up until 18 months, after which there is a regression in motor and language skills accompanied to behavioral and autonomic deficits. Diagnosis of RTT in males is rare as patients do not often survive past infancy (Reichow et al., 2015), except for rare cases reported beyond early childhood (Pitzianti et al., 2019; Takeguchi et al., 2020). Consequently, the majority of patients with RTT are females with a prevalence of one in every 10,000 births. RTT is caused by mutations in the *MECP2* gene (Amir et al., 1999) which codes for Methyl-CpG binding protein 2 (MeCP2), a protein that binds to methylated DNA to regulate transcription. MeCP2 also interacts with other important molecules by regulating microRNA (miRNA) (Wu et al., 2010). Although ubiquitously present, MeCP2 is expressed mostly in the brain, and it is an important regulator in brain development. Its altered functionality leads to neurodevelopmental deficits including impaired modulation of brain cell connectivity.

Fragile X syndrome affects one in every 4000 males, and one in every 7000 females. The symptoms in male patients with FXS are more severe than in females, and include developmental delays, behavioral and social deficits. FXS patients may show some degree of intellectual disabilities, while females may have normal intelligence to mild intellectual disability (Maurin et al., 2014; Dahlhaus, 2018).

Fragile X syndrome is caused by the silencing of the Fragile X Mental Retardation gene 1 (*FMR1*), which is associated to CGG repeat expansion in its 5′UTR region (Pieretti et al., 1991; Verkerk et al., 1991; Dahlhaus, 2018). As result of the expansion, the 5’UTR and the promoter of the *FMR1* gene are hypermethylated, *FMR1* expression is silenced and its encoded protein, the Fragile X Mental Retardation Protein (FMRP) is not expressed. FMRP is an RNA-binding protein and a component of ribonucleoprotein complexes involved in shuttling between nucleus and cytoplasm, transport along dendrites and association to polyribosomes (Maurin et al., 2014; Richter and Zhao, 2021). Indeed, FMRP modulates the subcellular localization (Xing and Bassell, 2013) and expression of thousands of its target mRNAs (Maurin and Bardoni, 2018; Richter and Zhao, 2021). The role of FMRP in translational regulation - being both repressor and enhancer of translation – is to date its most studied function (Bechara et al., 2009; Darnell et al., 2011; Maurin and Bardoni, 2018; Richter and Zhao, 2021).

Because of their monogenic origin, it has been possible for researchers to develop animal models to study RTT (Na et al., 2012; Pietri et al., 2013; Ezeonwuka and Rastegar, 2014; Cortelazzo et al., 2020) and FXS (Dahlhaus, 2018). These models provide the necessary framework to study the function of MeCP2 and FMRP and extrapolate mechanisms of action and putative targets. In addition, these animal models allow pre-clinical testing to set up therapeutic approaches. Many animal models are now available to study RTT, and several of them have the same mutation present in patients. Additionally, *Mecp2* mutant models can also be generated for studying MeCP2 overexpression or *MECP2* duplication syndrome (Collins et al., 2004; Na et al., 2012; Bodda et al., 2013). *Mecp2* duplication syndrome is marked by features of autism and it is distinct from typical RTT (Ramocki et al., 2009, 2010).

Multiple models for FXS have been generated in flies (Drozd et al., 2018), zebrafish (Vaz et al., 2019), rat (Kulkarni and Sevilimedu, 2020), and mice (Bakker et al., 1994; Mientjes et al., 2006; Dahlhaus, 2018). The mouse model is the most used, as it recapitulates the main phenotype of the disorder. The similarities are present even if silencing of the gene was obtained by a classical knockout (KO) approach and not by CGG expansion: the KO-1 (Bakker et al., 1994) with the neomycin cassette in the exon 5 of the *Fmr1*-gene and the KO-2 (Mientjes et al., 2006) that was generated from a conditional *Fmr1* KO by flanking the promoter and first exon of *Fmr1* with lox P site. In addition, two *Fmr1* knockin (KI) model mice have been generated and they reproduced sporadic missense mutations identified in the *FMR1* gene in FXS patients (Zang et al., 2009; Prieto et al., 2021).

Despite different molecular etiology and severity of clinical presentation, there are several overlapping symptoms between the disorders: intellectual disabilities, seizures, communication deficits, attention deficits, and defects in the skeletal apparatus. At the cellular level, the impairment of synaptic function and plasticity is recognized in both diseases.

These commonalities can be explained by the interplay between MeCP2 and FMRP, and by the common targets between both molecules. Recently, it was reported that MeCP2 expression is elevated in *Fmr1* KO mice cerebral cortex, while FMRP levels are reduced in mice mutants for *Mecp2* (Arsenault et al., 2020). This reciprocal relationship was confirmed using MeCP2 knockdown mouse N2A, and human HEK-293 cells lines (Arsenault et al., 2020). MeCP2 association with *FMR1* gene has been shown *in silico* (Bach et al., 2020). Moreover, MeCP2 and FMRP influence the expression of brain derived neurotrophic factor (BDNF), and, on the other hand, alteration in BDNF signaling affects the expression of *Fmr1* (Castrén and Castrén, 2014; Vicario et al., 2015).

Several authors, to clarify the neurobiology of RTT and FXS, investigated the molecular targets of MeCP2 and of FMRP (Skene et al., 2010; Darnell et al., 2011; Baubec et al., 2013; Maxwell et al., 2013; Gabel et al., 2015; Rube et al., 2016; Maurin et al., 2018; Sawicka et al., 2019). These studies show that many of the targets are involved in neurodevelopment and modulate brain function. With this premise, here we discuss the similarities and differences between mechanisms of synaptic function and plasticity in RTT and FXS as well as common molecular factors modulated in both disorders.

## Altered Synaptic Functioning in Rett Syndrome and Fragile X Syndrome

Alterations in synaptic function have been reported in both RTT and FXS (Sidorov et al., 2013; Bagni and Zukin, 2019; Banerjee et al., 2019) with functional consequences on the balance between excitation and inhibition (E/I ratio) and the mechanisms of synaptic plasticity (Table 1). The E/I ratio has been found to be altered in several neuropsychiatric disorders. While it represents a change in the extent of excitatory and inhibitory transmission, the underlying activity is complicated and multifaceted (Sohal and Rubenstein, 2019). Several sources report a shift in the E-I ratio in both RTT and FXS, however the alterations may differ depending on the brain region or cell-type investigated.

**TABLE 1:** Altered synaptic function in Rett syndrome and Fragile X syndrome.

Source	Cell type (Age)	Finding	References
*Mecp2^*Null/y*^*, Mecp2^*Tg1*^	Hippocampal neurons (7–14 DIV)	MeCP2 regulates glutamatergic synapse number and synapse strength	Chao et al., 2007
*Mecp2^–/Y^*	L5 neurons in S1 (P28–P35)	Reduced Cortical Activity: ↓mEPSC	Dani et al., 2005
*Mecp2^–/Y^*	L2/3 neurons of M1 (P21–28)	↓Local Excitatory input; No Change to Local Inhibitory input	Wood and Shepherd, 2010
*Mecp2^–/Y^*	L2/3 neurons of V1 (P∼45)	↓ Excitatory and ↓ inhibitory conductance; Altered GABA reversal potential	Banerjee et al., 2016
*Mecp2^–/Y^*	Pyramidal Neurons of the Hippocampus (P1, DIV 11–14)	↓ mEPSC; No change in mIPSC	Nelson et al., 2006
*Mecp2^–/Y^*	Layer 2/3 pyramidal neurons of S1 (P25–35). Primary culture of striatal neurons (P1, newborn)	↓ mIPSC	Chao et al., 2010
*Mecp2* ^*tm1.1Jae*^	Pyramidal CA3 Hippocampal Neurons (P40–60)	↑ mEPSC ↓mIPSC	Calfa et al., 2015
*Mecp2^–/Y^*	Layer 4 pyramidal and paravalbumin neurons of V1 (P28–30 and P50)	Selective ↓ excitatory input; No change in inhibitory or thalamo-cortical input	He et al., 2014
*Mecp2^–/Y^*	PV + and SOM + neurons	Specific RTT-like symptoms: ↓ Motor coordination and learning/memory (PV + *Mecp2^–/Y^*), seizures and ↑ Stereotypes (SOM + *Mecp2^–/Y^*)	Ito-Ishida et al., 2015
*Mecp2* ^*tm1.1Jae*^	Layer 5 pyramidal neurons of mPFC (p32–42)	↓ E/I Ratio and sEPSC; No change sIPSC, mEPSC, mIPSC	Sceniak et al., 2016
*Mecp2^–/Y^*	Layer 2/3 and layers 5/6 of V1 (P60–240)	↓ E/I Ratio; ↓ Excitatory input	Durand et al., 2012
*Mecp2*^30I^ heterozygous iPSCs	IPSCs differentiated into glutamatergic neurons	↓ Sodium and potassium currents ↓ sEPSC frequency	Farra et al., 2012
*Fmr1* ^–/Y^	Layer 2/3 neurons of S1 (P19–31)	↓ Inhibitory control on pyramidal output	Paluszkiewicz et al., 2011
*Fmr1^–/Y^*	Principal excitatory neurons of Basolateral amygdala (P21–30)	↓ Tonic GABAergic capacity; No change in E/I ratio	Martin et al., 2014
*Fmr1^–/y^, Fmr1^–/HET^*	Pyramidal neurons of CA1 (P18–23)	↑ E/I ratio; ↓GABA release (TA inhibitory synapses)	Wahlstrom-Helgren and Klyachko, 2015
*Fmr1^–/Y^*	Layer 2/3 and layer 4 fast-spiking inhibitory and excitatory (mostly spiny stellate), Neurons of somatosensory cortex	↓ Excitatory input onto fast spiking Inhibitory neurons and onto excitatory neurons	Gibson et al., 2008
*Fmr1* ^–/Y^	CA1 pyramidal layer of dorsal hippocampus	↑ Theta oscillation power; ↑ Slow gamma band coherence; ↓ Spike count of specific interneurons	Arbab et al., 2018
*Fmr1^–/Y^*	Layer 4 fast-spiking neurons, layer 5 fast-spiking and layer 4 excitatory neurons in the somatosensory cortex	↓ Excitatory input on fast-spiking inhibitory neurons	Patel et al., 2013

*Main findings of studies reporting on synaptic function in Rett syndrome and Fragile X syndrome. *Mecp2*, methyl-CpG binding protein 2 gene; S1, primary somatosensory cortex; M1, primary motor cortex; V1, primary visual cortex; mEPSC, miniature excitatory postsynaptic currents; mIPSC, miniature inhibitory postsynaptic currents; DIV, days *in vitro;* PV+, parvalbumin-positive neurons; SOM+, somatostatin-positive neurons; mPFC, medial prefrontal cortex; sEPSC, spontaneous excitatory postsynaptic currents; sIPSC, spontaneous excitatory postsynaptic currents; iPSC, induced pluripotent stem cells; *Fmr1*, fragile X mental retardation 1 gene; TA, temporoammonic.*

In symptomatic *Mecp2* KO mice there is a shift in E/I ratio in favor of inhibition present in a number of brain regions including S1, V1, and mPFC (Dani et al., 2005; Durand et al., 2012; Sceniak et al., 2016). However, at birth *Mecp2* mutant mice show an increased glutamatergic transmission, due to the altered shift in GABA function, which is excitatory during the first phases of development, but reverses during the first weeks of postnatal development. In *Mecp2* KO mice the GABA shift is postponed, and treatment with bumetanide, an inhibitor of the chloride channel NKCC1 reduces the effects of the delay. When bumetanide is administered prenatally, some of the symptoms of RTT are decreased, but the respiratory dysfunction and the mortality remain (Lozovaya et al., 2019). Symptomatic *Mecp2* KO mice display decreased dendritic spine density (Kishi and Macklis, 2004; Tropea et al., 2009; Castro et al., 2014) and MeCP2 deficit has been found to decrease glutamatergic synapse number and strength (Chao et al., 2007).

Deletion of *Mecp2* in particular cell neuronal subtypes in mice, reveal the complicated nature of shifts in E/I ratio. Conditional deletion of *Mecp2* in parvalbuminergic (PV) neurons removes the experience dependent critical period of plasticity in the visual cortex, and produces only a partial Rett-like phenotype, specifically motor dysfunction (He et al., 2014; Ito-Ishida et al., 2015). A more general effect of GABAergic neurons has been observed in the brainstem, with a reduction in the number of GABAergic synapses. In patients with RTT, abnormality of inhibitory transmission is associated with respiratory dysfunction, and blockade of GABA reuptake decreased the breathing dysfunction in Mecp2 KO mice (Abdala et al., 2016).

Studies in patients-derived cells confirm the decrease excitatory synaptic transmission in iPS cells derived from mice. These results were also observed in mouse preparations *in vivo* and *in vitro* (Farra et al., 2012).

In FXS mouse models changes to E/I ratio appear to be more specific compared to the *Mecp2* KO mice. Increased intrinsic excitability is observed in FXS at cellular, circuit and behavioral level. The cellular excitability stays with altered ion channels activity, dependent both on the translational activity of FMRP, but also on the direct interaction between FMRP and ion channels (Contractor et al., 2015). In FXS, one aspect of hyperexcitability is linked to the delayed switch in GABA polarity. As for RTT, this timeline is delayed in FXS, where the GABA transmission remains excitatory for longer. The overall increased excitability of the circuits influences also synaptic excitability and spike-timing dependent plasticity, and it is dependent on the chloride transporter NKCC1.

Another aspect of the increased excitability in FXS is due to a reduced expression of GABAA receptors’ subunits, with reduced frequency of IPSCs, but not amplitude of GABA currents. The general imbalance in favor of excitation in FXS reflects on the hyper-reactivity to stimuli, anxiety, and seizures in animal models and patients.

Studies in human-derived cells confirmed the morphological findings in FMRP deprived cultures, but they not always confirmed the altered excitability, especially at early stages of development (Telias et al., 2015). This discrepancy can be due to an un-matched decrease in FMRP expression during the *in vitro* development (Linda et al., 2018).

The frequency of the brain waves is also affected in FXS. In *Fmr1* KO mice, increased synchronization of local field potentials occurs and may underlie deficits to information processing in hippocampal circuitry. This hyper-synchronization was characterized in a freely moving mouse, as increased theta power and coherence of slow gamma oscillations (Sohal et al., 2009; Arbab et al., 2018). More recently, the single-cell analyses of a subpopulation of interneurons in *Fmr1*-KO brain at Post-natal day (PND) 18 highlighted the increased levels of inhibitory markers in the absence of FMRP (Castagnola et al., 2020).

### Morphological Correlates of Synaptic Functions

Alterations in synaptic functions are reflected also in morphological differences between the two disorders. Reduced neuronal cell size and numbers of dendrites have been observed in patients with RTT alongside short dendrites and a decrease in dendritic spines (Armstrong, 2005; Belichenko et al., 2008). In RTT, there is an overall decrease in brain volume differences and altered brain structures that are also present in mice studies (Chen et al., 2001; Ballas et al., 2009; Xu et al., 2014).

In contrast to RTT and MeCP2 deficiency, a lack of FRMP is associated with increases spine density in human patients and mice models (Levenga et al., 2011; Hodges et al., 2017). Both in *Fmr1* KO mice, and in brain from patients, it has been reported an increase in the number of elongated spines (filopodia) in absence of functional FMRP (Comery et al., 1997), suggesting a reduction in the pruning of spines (Irwin et al., 2000). However, recent studies on spine functionality (Thomazeau et al., 2020) and spine compartmentalization over time (Wijetunge et al., 2014), find that the altered morphology does not correlate with abnormal function between *Fmr1* KO mice and controls. These findings challenge the view of immature status of connections in FXS (He and Portera-Cailliau, 2013), and even the turnover of the spines seems to be invariant to that of matched controls. However, all these measures can be dependent on the brain area and on the stage of development (He and Portera-Cailliau, 2013).

In fact, the presence of the filopodia is dependent on the stage of development and on the preparation. Nimchinsky et al. (2001) analyzed mutant and control mice at several developmental stages and showed that differences in spine morphology and density decreased between 1 and 4 weeks of age. The closing of these phenotypic gaps originally suggested that FMRP played a role in coordinating synaptogenesis in a time-dependant manner. Different cell populations of the hippocampus also show that long and irregular spines are also present in juvenile and adult *Fmr1* KO mice, while *in vitro* and *in vivo* studies show discrepancies for spine densities as with LTP and LTD function (as reviewed by Bostrom et al., 2016). A summary of the studies investigating morphological correlates of synaptic functions in RTT and FXS is reported in Table 2.

**TABLE 2:** Morphological correlates in Rett syndrome and Fragile X syndrome.

Source	Region/Sample	Experiment	Findings	References
*Mecp2^*tm1.1Jae*^*, *Mecp2^308/Y^*, *Mecp2^*tm1.1Bird*^*, *Patients with RTT*	Hippocampus, cortex, cerebellum	Imaging studies	↓ Neuronal size; ↓ Dendrite numbers; ↓ Dendrite length; ↓ Dendrite spines	Armstrong, 2005; Belichenko et al., 2008
*Mecp2^–/y^, Patients with RTT*	Cerebral cortex, total brain	Imaging studies	↓ Volume	Chen et al., 2001; Armstrong, 2005
*Fmr1* ^–/Y^	Hippocampus (25 weeks)	Immunohistochemistry, imaging studies	↑ Spine density	Levenga et al., 2011; Hodges et al., 2017
*Fmr1^–/Y^, Patients with FXS*	Cortex (16 weeks), temporal cortex	Immunohistochemistry, imaging studies	↑ Filopodia; ↑ Dendrite length; ↑ Dendrite spines	Comery et al., 1997; Irwin et al., 2000
*Fmr1* ^–/Y^	CA1 and layer 5 neurons (P14-P37), hippocampal slices (P25-P35)	Imaging studies	Altered morphology may not correlate with abnormal function	Wijetunge et al., 2014; Thomazeau et al., 2020
*Fmr1* ^–/Y^	Layer 5 neurons (P7-P28)	Imaging studies	Synaptogenesis is mediated by FMRP in a time-dependant manner	Nimchinsky et al., 2001

*Main findings of studies reporting on morphological phenotypes in Rett syndrome and Fragile X syndrome. *Mecp2*, methyl-CpG binding protein 2 gene; RTT, Rett syndrome; *Fmr1*, fragile X mental retardation 1 gene, FXS, Fragile X syndrome.*

## Common Forms of Synaptic Plasticity Disrupted in Rett Syndrome and Fragile X Syndrome

The long-term activity-dependent variation in synaptic connectivity and the associated molecular changes are defined as synaptic plasticity (Citri and Malenka, 2008). Several forms of synaptic plasticity have been reported to be disrupted in RTT and FXS (Huber et al., 2002; Blackman et al., 2012; Wang et al., 2012; Na et al., 2013; Wondolowski and Dickman, 2013).

### Alterations in Long-Term Potentiation and Long-Term Depression in Rett Syndrome and Fragile X Syndrome

Both RTT and FXS display endophenotypes that signal aberrant Long-term potentiation (LTP) and long-term depression (LTD), which are Hebbian Forms of plasticity.

In RTT, deficits have been reported in excitatory synapses and LTP. Hippocampal slices show reduced LTP in *Mecp2* KO (Asaka et al., 2006) and *Mecp2*^308/Y^ mice (Moretti et al., 2006). Reduced potentiation is present around onset of symptoms while pre-symptomatic mice maintain the same level of activity observed in matching controls, suggesting that synaptic dysfunction and decline in plasticity is an early event in RTT. Pyramidal neuron synapses in the hippocampus are indeed potentiated in *Mecp2* KO mice, however there is a failure to regulate AMPA receptors post-activation and an overall deficit in LTP (Li et al., 2016). *Mecp2* KO mice display a lack of structural plasticity, and enlarged spines are observed regardless of stimulation or sham (Li et al., 2016). AMPA receptor-related transmission is enhanced at hippocampal synapses and over time, the decrease in internalization fails to counterbalance excessive accumulation. In addition, the lack of receptor trafficking prevents activated synapses from becoming plastic, affecting both LTP and LTD.

Long-term depression is also altered in RTT, however to a lesser extent than LTP. Moretti et al. (2006) induced LTD with two different stimulus paradygms in the hippocampus of *Mecp2*^308/Y^ mice and show that when LTD was induced by administration of the mGluR agonist 3,5-dihydroxyphenylglycine (DHPG), the response was comparable in mutant and wildtype mice. However, when paired pulse stimulation was applied, LTD was observed in wild type mice, but not in *Mecp2*^308/Y^ mice. Since both stimulation paradigms affect the post-synaptic sites, but only the paired pulse stimulation protocol is dependent on the presynaptic site, the authors conclude that the impairments in plasticity are due to altered presynaptic terminals, while there is some preservation of LTD. It has also been shown that hippocampal slices of symptomatic mice with an *Mecp2* KO show no NMDAR-dependent LTD by low frequency stimulation (Asaka et al., 2006). More recently, in younger P15 *Mecp2* KO mice, LTD has been induced by DHPG at two different stages: early and late (Lozovaya et al., 2019). Only early induced LTD displays a significant decrease in the amplitude of the response, implicating even earlier synaptic impairment onset in RTT mice.

In FXS LTP was reported to be reduced in several brain regions (Desai et al., 2006; Lauterborn et al., 2007; Suvrathan and Chattarji, 2011; Seese et al., 2012), however, the main form of plasticity studied in FXS is LTD.

The first to show alterations in synaptic plasticity in the absence of FMRP were Huber and colleagues (Huber et al., 2002), who showed an increase in mGluR-dependent LTD in the hippocampus of *Fmr1* KO mice. The dysregulation of mGluR signaling was further confirmed by other authors (Weiler et al., 1997; Gross et al., 2012; Tian et al., 2017), and all the data support the theory that FMRP controls the translation of specific proteins involved in synaptic function, including glutamatergic receptors.

Similarly to what reported in RTT, AMPA aberrant receptors trafficking is driven by a lack of FMRP and consequently associated with cognitive deficits (Nakamoto et al., 2007). PSD-95 synthesis increases in response to DHPG-activation of mGluR, and is co-translated with FMRP (Todd et al., 2003). Further studies in other brain areas showed that LTD in enhanced in multiple cell populations, including cerebellum, where it controls abnormal motor behavior and development of synaptic circuitry in the somatosensory cortex (Greenough et al., 2001; Huber et al., 2002; Koekkoek et al., 2005).

Overall, LTP deficits have been shown in RTT and to a lesser extent in FXS. On the other hand, FXS tends to display enhanced LTD, while RTT shows some LTD preservation.

A summary of the experiments exploring Hebbian forms of plasticity in RTT and FXS is reported in Table 3. Beside LTP and LTD, other forms of plasticity are affected in RTT and FXS.

**TABLE 3:** Alteration of long-term potentiation and long-term depression in Rett syndrome and Fragile X syndrome.

Source	Region/Sample	Experiment	Findings	References
*Mecp2*^–/Y^ established by crossing two lines (Chen et al., 2001; Guy et al., 2001)	Hippocampal slices	Tetanic stimulation, theta-burst stimulation, low frequency stimulation	↓ NMDA- dependant LTP; ↓ NMDA- dependant LTD in symptomatic mice; No difference in presymptomatic mice	Asaka et al., 2006
*Mecp2* ^308/Y^	Hippocampal slices	DHPG LTD conduction, theta-burst stimulation, paired-pulse facilitation	↑ STP; ↓ LTP; No changes in LTD	Moretti et al., 2006
*Mecp2^–/Y^*	Hippocampal slices (P20–P22, P45–P65)	Theta-burst stimulation, forskolin-induced chemical LTP	↓ LTP	Li et al., 2016
*Mecp2^–/Y^*	CA3 pyramidal layer (P14–P16)	DHPG LTD Induction, maternal pretreatment of bumetanide	↓ Early-LTD; No difference in late-LTD; Bumetanide improves early-LTD	Lozovaya et al., 2019
*Fmr1^*exon8*–*KO*^* by CRISPR/Cas9	Rat hippocampi (8–12 weeks)	Theta-burst stimulation, DHPG LTD Induction, low frequency stimulation	↑ LTD by DHPG LTD induction; ↓ LTD by low frequency stimulation; ↓ LTP	Tian et al., 2017
*Fmr1^–/Y^*	Hippocampal slices (P21–30)	Paired-pulse low-frequency stimulation, NMDAR-LTD induction	↑ mGluR-dependant LTD; No difference in NMDAR-LTD or LTP	Huber et al., 2002
*Fmr1^–/Y^* and Purkinje cell-specific *L7-Fmr1^–/Y^*	Cerebellar slices	Low frequency stimulation	↑ LTD	Koekkoek et al., 2005
*Fmr1^–/Y^*	Hippocampal slices	High frequency stimulation	No difference in LTP	Godfraind et al., 1996
*Fmr1^–/Y^*	Hippocampal slices (P35–P56)	Paired-pulse facilitation, theta-burst stimulation	No difference in LTP	Paradee et al., 1999
*Fmr1^–/Y^*	Somatosensory cortex (P5–P11)	LTP stimulation, bumetanide treatment	↑ LTP which is corrected by bumetanide	He et al., 2019

*Main findings of studies reporting on long-term potentiation and long-term depression in Rett syndrome and Fragile X syndrome. *Mecp2*, methyl-CpG binding protein 2 gene; NMDA, *N*-methyl-D-aspartate; LTP, long-term potentiation; LTD, long-term depression; DHPG, 3,5-dihydroxyphenylglycine; STP, short-term potentiation; *Fmr1*, fragile X mental retardation 1 gene; NMDAR, *N*-methyl-D-aspartate receptor.*

### Alterations in Homeostatic Plasticity in Rett Syndrome and Fragile X Syndrome

Homeostatic plasticity controls the changes in synaptic strength that individual neurons operate in response to prolonged changes of neuronal stimulation, and it also mediates the balance of excitation and inhibition. Both RTT and FXS have shown alterations in mechanisms of homeostatic plasticity, and these alterations may explain the general changes in neuronal activity in patients and animal models with RTT and FXS (Table 4).

**TABLE 4:** Altered homeostatic plasticity in Rett syndrome and Fragile X syndrome.

Source	Region/Sample	Experiment	Findings	References
Mecp2^–/y^ by shRNA	Hippocampus	Whole cell voltage clamp	*Mecp2* downregulation prevents activity-dependent synaptic scaling	Qiu et al., 2012
Mecp2 knockdown by shRNA	Rat visual cortex neurons	Whole cell patch clamp	MeCP2 is necessary for cell-autonomous scaling up	Blackman et al., 2012
*Mecp2* ^*tm1.1Jae*^	Hippocampal neurons	Whole cell patch clamp and molecular analysis	↑ EEA1 expression re-establishes synaptic scaling in *Mecp2* mutant mice	Xu and Pozzo-Miller, 2017
*Mecp2*^*S421A; S424A/y*^ and Mecp2^–/y^	Hippocampal neurons	Whole cell patch clamp and molecular analysis	MeCP2 phosphorylation is necessary for synaptic scaling	Zhong et al., 2012
*Fmr1^–/Y^*	Hippocampal slice/cultures	Patch Clamp	FMRP is necessary postsynaptically to mediate the RA mediated synaptic scaling	Soden and Chen, 2010
*Fmr1^–/Y^*	Hippocampal neurons	Patch Clamp	GluA1 ubiquitination synaptic downscaling is prevented in *Fmr1* knockout mice	Lee et al., 2018

*Main findings of studies reporting homeostatic plasticity in Rett syndrome and Fragile X syndrome. *Mecp2*, methyl-CpG binding protein 2 gene; *Fmr1*, fragile X mental retardation 1 gene, FRMP, fragile X mental retardation protein EEA1, early endosome antigen 1, RA, retinoic acid.*

One of the main mechanisms controlling homeostatic changes is synaptic scaling, and it relies on the synthesis, trafficking, and function of AMPA receptors.

Defects in functional levels of *Mecp2* alter homeostatic plasticity. Hence, in RTT, where there is a loss of function of MeCP2, there is impaired ability to respond to homeostatic changes (Na et al., 2013). One of the first studies of homeostatic plasticity in RTT was performed in neuronal cultures. In this study, stimulation with bicuculline produced an increase in neuronal activity, with a sequential decrease in the expression of the AMPA receptors subunit GluR2 and a reduction of mEPSC amplitude. However, the expression of GluR2 is under the control of the transcriptional repressor MeCP2, whose levels also increase during bicuculline stimulation. As a result, the bicuculline treatment in *Mecp2* KO neurons, does not lead to the expected decrease of the GluR2 receptor subunit at the synapse. These experiments were among the first to show the importance of Mecp2 in the control of synaptic scaling (Qiu et al., 2012), and they were confirmed by Blackman et al. (2012), who showed that reduced neuronal drive in *Mecp2* KO preparations does not produce the expected increase in synaptic scaling as observed in WT controls. One of the factors controlling homeostatic plasticity in RTT is the Early Endosome Antigen 1 (EEA1), which regulates AMPA receptor endocytosis. EEA1 expression is reduced in *Mecp2* KO mice, where the synaptic scaling is reduced. However, the increased expression of EEA1 in *Mecp2* KO cultures, reinstates the ability to scale the synapses in response to changes in activity (Xu and Pozzo-Miller, 2017). It is important to remember that not only *Mecp2* expression, but also its activation, is important for the proper functioning of the protein. Indeed, post translational modifications of MeCP2 have also been found to be implicated in synaptic function and homeostasis (Bellini et al., 2014) and the phosphorylated MeCP2 modulates synaptic scaling -down through mGluR5 (Zhong et al., 2012).

Fragile X mental retardation protein has been involved in synaptic scaling in response to both increased and decreased activity- as observed in *Fmr1* KO mice and in neuronal cultures derived from patients with FXS. The increased activity of AMPA receptors consequent to a decrease in activity, is mediated by the Retinoic Acid (RA), which is produced in response to the altered activity, and promotes the synthesis of new AMPA receptors. However, in FXS, while the transcription of the AMPA receptor remains unchanged, the RA- mediated translation of AMPA is reduced, and only restoration of the proper full length functional FMRP re-establishes synaptic scaling in primary cultures derived from *Fmr1KO* mice (Soden and Chen, 2010). These results suggest that FMRP is essential for the postsynaptic response in RA-mediated synaptic scaling. However, RA is required for the combined action of TTX and NMDA blockade- as there are no changes in scaling with TTX alone. In this regard it is interesting to note that the RA does not affect spine morphology and number. Zhang and colleagues confirmed the impaired synaptic scaling in neuronal cultures derived from patients with FXS (Zhang et al., 2018) and the role of RA as mediator of synaptic scaling. The RA action is effective both on excitatory and inhibitory synapses. Interestingly, RA is one of the mediators of the post- to presynaptic communication and could be involved in linking the post-synaptic events to pre-synaptic adjustments. In FXS, the imbalance in protein synthesis determines a dysregulation in RA which is important at postsynaptic level, but also at the presynaptic level with the regulation of EPSC frequencies (Wang et al., 2011; McCarthy et al., 2012).

Synaptic downscaling – the scaling down in response to increased synaptic activity- is also altered in *Fmr1* KO mice. One of the mechanisms of downscaling is the degradation of AMPA receptor through ubiquitination (Lee et al., 2018). The ubiquitination of AMPA receptors is mediated by a complex cascade of molecules, which includes cell-cycle molecules and phosphatases, and FMRP interferes with the mechanism that leads to homeostatic-dependent ubiquitination.

It is worth of note that the Neuroligin-Neurexin complexes, involved in circuitry development and function is also essential for presynaptic homeostatic plasticity (Sons et al., 2006). Several molecular studies in RTT and FXS report that these molecules are targets of FMRP and MeCP2 (Darnell et al., 2011; Gulmez Karaca et al., 2018; Maurin et al., 2018; Raman et al., 2018), suggesting that impaired homeostatic plasticity in these syndromes may be mediated by deficits in Neuroligin or Neurexin.

## Molecules and Pathways Controlling Changes in Synaptic Strength and Connectivity in Rett Syndrome and Fragile X Syndrome

The alteration in several forms of plasticity observed in RTT and FXS can be explained considering that many molecular targets of MeCP2 and FMRP are involved in the regulation of synaptic function. The identification of these molecular regulators can shed light on the neurobiology of RTT and FXS and can suggest strategies for treatment. In this section we will examine several of these molecules and pathways (Table 5).

**TABLE 5:** Pathways implicated in Rett syndrome and Fragile X syndrome.

Source	Region/Sample	Experiment	Findings	References
**Brain-derived neurotrophic factor signaling pathway**				
*Mecp2^–/ Y–,^* Conditional BDNF-over-expression	CA2 neurons	Behavioral assessment, electrophysiology, immunohistochemistry	BDNF overexpression reverses some RTT phenotypes	Chang et al., 2006
*Patients with RTT*	Serum and CSF	Patient studies	No changes in BDNF expression in patients with RTT vs. healthy controls	Vanhala et al., 1998
*Mecp2^–/y^, patients with RTT*	Cerebrum, frontal cortex, whole brains (mice)	Chromatin immunoprecipitation, qPCR	↓BDNF expression↑; TrkB	Abuhatzira et al., 2007; Deng et al., 2007
*Mecp2^–/y^*	Hippocampal glutamatergic neurons, CA1	Immunofluorescence	↓PSD95; (1–3)IGF1 restores PSD95 levels	Chao et al., 2007; Tropea et al., 2009
*Fmr1^–/y^*	Hippocampal neurons (P0)	Western blotting	↓PSD95 localization; restored by inhibition of mTORC-S6K1 signaling	Yang et al., 2019
*Fmr1^–/y^*	Primary cortical cultures (14–15DIV; E15), hippocampal neuronal cultures (E19)	Immunofluorescence, western blotting, mRNA stability assay	↓PSD95; FMRP binds to and stabilize Psd95 mRNA	Todd et al., 2003; Zalfa et al., 2007
**Insulin-like growth factor 1 signaling pathway**				
*Mecp2^–/y^*	CSF	Patient studies	No changes in IGF1 expression	Riikonen, 2003
*Mecp2^–/y^, Mecp2^–/+^*	Motor cortex, cortical slices	Behavioral assessment, immunocytochemistry, electrophysiology	IGF1 improves several RTT symptoms incl. cortical plasticity	Tropea et al., 2009; Castro et al., 2014; Khwaja et al., 2014; O’Leary et al., 2018
*Fmr1^–/y^*	Testes	Western blot, *Igf1r* knockout	Correcting IGF1R levels reduces macro-orchidism	Wise, 2017
*Fmr1^–/y^, Patients with FXS*	Mice (14 weeks), primary hippocampal cell cultures (17 DIV)	Clinical trials, behavioral assessment, kinase assays	NNZ-2566 administration improves patients’ symptom scoring	Deacon et al., 2015; Berry-Kravis et al., 2020
**Cyclic adenosine monophosphate (cAMP) response element binding protein signaling pathway**				
*MECP2T^158M/T158M^ hESC, MECP2- V247fs-MT iPSC, Mecp2^–/+^*	hESC differentiated into forebrain neurons, iPSC	Electrophysiology, western blotting, behavioral assessment	↓CREB; Correcting CREB levels improves some RTT phenotypes	Bu et al., 2017
*SWR/J mice, dfmr1*^3/+^ Drosophila	SWR/J mice (15–25 weeks,	qPCR, western blotting, immunofluorescence, behavioral assessment	*Fmr1* is bound by CREB	Kanellopoulos et al., 2012; Rani and Prasad, 2015
**Phosphatidylinositol-3-kinases signaling pathway**				
*Mecp2^308^, Mecp2* ^*tml.lJae*^	Male (*Mecp2^308^*, 5 months), cortical neurons (P1)	Behavioral assessment, qPCR, western blotting, immune-histochemistry	↓PI3K pathway activation	Ricciardi et al., 2011; Yuan et al., 2020
*Fmr1^–/y^*	Primary hippocampal neurons (E17)	Western blotting, immune-histochemistry, kinase assays	↑PI3K pathway activation	Gross et al., 2010
*Fmr1^–/y^*	Cortex, cerebellum (P11–13), hippocampus (P28–32) Hippocampal slices (4–6 weeks)	Immuno-histochemistry, bioinformatics, qPCR, electrophysiology	↑mTOR phosphorylation	Sharma et al., 2010; Casingal et al., 2020
**Mitogen-activated protein kinase signaling pathway**				
*Mecp2^–/y^*	Motor cortex, cortical slices	Behavioral assessment, immuno-cytochemistry, electrophysiology	↓MAPK activation; rhIGF1 increases activation	Castro et al., 2014
*Fmr1^–/y^*	Mice (14 weeks), primary hippocampal cell cultures (17 DIV)	Behavioral assessment, kinase assays	↑MAPK activation; Corrected by NNZ-2566	Deacon et al., 2015
**Bioenergetics**				
Patients with RTT	34 patients with RTT, 37 healthy controls	Metabolomic analysis	Metabolic dysfunction, oxidative stress.	Neul et al., 2020
*Mecp2^*tm1.1Bird*^, Patients with RTT*	Isolated microglia, primary hippocampal cell cultures, fibroblasts isolated from patients with RTT	Immunofluorescence, qPCR, imaging studies, western blotting, bioenergetic assays	↓Microglial viability; ↓Microglia numbers; ↑ROS; ↓ATP production; ↑Glutamate	Jin et al., 2015; Crivellari et al., 2021
*Fmr1^–/y^*	Fibroblasts cell lines, synaptosomes, primary hippocampal (E19) and cortical cell cultures (P0–P2)	Behavioral assessment, immune-precipitation, western blotting, qPCR, bioenergetic assay	Mitochondrial proton leak	Licznerski et al., 2020
*Fmr1^–/y^*	Brain slices, macrophages, total brain; (2–6 months)	Bioenergetic assay,	↑ROS; ↑Lipid peroxidation; ↑Protein oxidation; ↑NADPH oxidase activity	El Bekay et al., 2007
*Fmr1^–/y^*	Various	Review	↑Metabolites from superoxide attack on lipids ↑ROS	Maurin et al., 2014

*Main findings of studies reporting signaling and pathway dysfunctions in Rett syndrome and Fragile X syndrome. *Mecp2*, methyl-CpG binding protein 2 gene; BDNF, brain-derived neurotrophic factor; RTT, Rett syndrome; CSF, cerebrospinal fluid; TrkB, tropomycin receptor kinase B; *Fmr1*, fragile X mental retardation 1 gene, PSD95, postsynaptic density protein 95; DIV, days *in vitro*; FRMP, fragile X mental retardation protein; IGF1, insulin-like growth factor 1; *Igf1r*/IGF1R, insulin-like growth factor 1 receptor; NNZ-2566, (1–3)IGF1 tripeptide; CREB, cAMP response element binding protein; hESC, human embryonic stem cells; iPSC, induced pluripotent stem cells; PI3K, phosphatidylinositol-3-kinase; mTOR, mammalian target of rapamycin; MAPK, mitogen-activated protein kinase; rhIGF1, recombinant human insulin-like growth factor 1; ROS, reactive oxygen species.*

Brain derived neurotrophic factor (BDNF) controls brain development and function, and it is involved in activity dependent plasticity. Its expression is highly specific and functionally defined in the mammalian brain (Kowiański et al., 2018). BDNF expression is dependent on the neurodevelopmental stage, and it is present in several different forms that bind different receptors. Pre-Pro-BDNF is concentrated at the endoplasmic reticulum before becoming pro-BDNF at the Golgi apparatus (Foltran and Diaz, 2016). Pro-BDNF is highly expressed during early postnatal development and binds p75 Neurotrophin Receptor (p75NTR) and Sortilin receptor, with a particular polymorphism Val66Met dictating the receptor binding properties (Anastasia et al., 2013). Meanwhile the mature BDNF protein (mBDNF) is present more during adulthood and binds the tyrosine kinase B receptor (TrkB) (Reichardt, 2006).

All these receptors are located on the membranes and in intracellular compartments. The complex resulting from pro-BDNF, Sortilin receptor, and p75NTR signals to RhoA, NFKB and JNK related pathways. These pathways have roles in neurodevelopment, survival and apoptosis respectively (Reichardt, 2006). mBDNF binding to TrkB results in receptor dimerization and phosphorylation on membrane lipid rafts. This complex is also associated with a number of downstream signaling pathways including PI3K, mitogen-activated protein kinase (MAPK), PLC-gamma and GTPases of the Rho family all with a range of neuronal functions (Suzuki et al., 2004). The amount of neuronal cellular processes that BDNF effects means that its expression is highly relevant to synaptic plasticity and functioning. Indeed decrease in BDNF concentration inhibits synaptogenesis and dendritic arborization (Wang et al., 2015).

Brain derived neurotrophic factor administration in control mice increases the synaptic localization of PSD95. This effect is suppressed in *Fmr1* KO mice, but it can be retrieved by inhibition of mTORC-S6K1 signaling (Yang et al., 2019). PSD95 is one of the core postsynaptic proteins that functions by regulating activity of excitatory neurotransmitter receptors (Keith and El-Husseini, 2008), and its expression is modulated by FMRP (Muddashetty et al., 2011; Xing and Bassell, 2013; Ifrim et al., 2015).

Brain derived neurotrophic factor level is deficient in *Mecp2* KO mouse brain and, consistently, its overexpression has been shown to reverse some RTT phenotypes (Chang et al., 2006). In patients with RTT, BDNF serum and cerebrospinal fluid (CSF) protein levels have been found to be no different from healthy controls (Vanhala et al., 1998), while in the brain the BDNF level is decreased and TrkB level is increased (Abuhatzira et al., 2007; Deng et al., 2007).

### Insulin-Like Growth Factor 1

Another factor influencing the intracellular pathways controlling synaptic strength is IGF1, which is a protein involved in growth, maturation, and neuronal development. Although CSF levels of Insulin-Like Growth Factor 1 (IGF1) have been found to be unchanged in RTT (Riikonen, 2003), both the full IGF1 molecule, and its functionally active cleavage product (1-3)IGF1 have been shown to ameliorate symptoms of the RTT pathophysiology (Tropea et al., 2009; Castro et al., 2014; Khwaja et al., 2014; O’Leary et al., 2018). IGF1 and (1-3)IGF1 administration in a RTT mouse model increases PSD95, dendritic arborization and excitatory current. Interestingly, these treatments also appear to re-establish cortical plasticity in *Mecp2* KO mice to levels observed in controls (Tropea et al., 2009; Castro et al., 2014). IGF1 signaling occurs primarily through PI3K-AKT and MAPK pathways (Fernandez and Torres-Alemán, 2012). IGF1R activates PI3K-AKT functions to increase neuronal survival (Dudek et al., 1997), while prolonged administration of IGF1 with growth hormone, determines pro-inflammatory responses via activation of MAPK (Wolters et al., 2017). IGF signaling to a lesser extent has been implicated in FXS, where correcting the low level of IGF1R was found to reduce macro-orchidism (enlarged testes) a phenotype characterizing all the adult male patients with FXS (Wise, 2017). More promising evidence comes from the use of the (1-3)IGF1 analog, NNZ-2566 (Trofinetide) as a treatment for FXS; in fact mouse models of FXS treated with NNZ-2566 showed improvements in cognitive function and hyperactivity. Also patients with FXS treated with NNZ-2566 improved in a number of clinical scoring tools (Deacon et al., 2015; Berry-Kravis et al., 2020), but further clinical tests are required to confirm the benefits of the treatment.

Both BDNF and IGF1 signals involve the activation of intracellular pathways involving the PI3K and MAPK cascades, which are related to both RTT and FXS and are involved in activity-dependent plasticity.

### Cyclic Adenosine Monophosphate Response Element Binding Protein

Cyclic adenosine monophosphate (cAMP) response element binding protein is involved in transcriptional changes induced by synaptic plasticity, including increasing neuronal excitability and synapse strengthening associated to LTP induction (Caracciolo et al., 2018). Transcriptional genes expressed by CREB activation include *c-Fos*, whose protein is linked to memory and learning (Gallo et al., 2018), and the co-localization of CREB and c-Fos is associated with long term synaptic plasticity (Miyashita et al., 2018). This molecule has been found to be decreased in *Mecp2* KO mice and rectifying CREB levels can correct some of the RTT symptoms (Bu et al., 2017). The *Fmr1* gene is thought to be bound and therefore regulated by CREB (Kanellopoulos et al., 2012; Rani and Prasad, 2015), however the relationship of CREB signaling to FXS synaptic plasticity requires further investigation, especially considering the role that FMRP has in the modulation of cAMP and cGMP levels, two molecules upstream the CREB expression (Delhaye and Bardoni, 2021). It was shown that In the hippocampus, Cilostazol (an inhibitor of Phosphodiesterase 3) increases the levels of c-fos and of insulin-like growth factor 1 (IGF-1) (Zhao et al., 2010) and activates CREB in PC12 cells (Zheng and Quirion, 2006). This result suggests a link between the levels of cAMP and cGMP – both targets of PDE3 – and the levels of IGF-1. Remarkably, the inhibition of PDEs both in FXS (PDE2, PDE4, PDE4D) and in RETT (PDE4) has been shown to improve socio-cognitive deficits in animal models and in patients.

### Phosphatidylinositol-3-Kinases

Phosphatidylinositol-3-kinases (PI3K) are a family of intracellular signaling molecules functioning downstream of G protein coupled receptors and tyrosine kinases.

Once activated, PI3K subsequently phosphorylates AKT which regulates cell cycle and apoptosis (Chalhoub and Baker, 2009). Further downstream of AKT phosphorylation, is the mammalian Target of Rapamycin (mTOR) whose activation regulates nutrition, energy sensing and growth (Zoncu et al., 2011). In *Mecp2* KO mice the activation of the PI3K/AKT/mTOR pathway is reduced, while in FXS it is increased. The alterations in PI3K/AKT/mTOR level in *Mecp2* KO mice can be restored using IGF1, and treatment with PI3K antagonists rescue FXS defects (Gross et al., 2010; Ricciardi et al., 2011; Yuan et al., 2020).

Alterations of FMRP levels affect the mGluR-PI3K-AKT-mTOR cascade. In normal conditions mGluR-dependent LTD requires rapid translation of dendritic mRNA, but in *Fmr1* KO mice LTD it is enhanced and insensitive to inhibition of protein synthesis (Huber et al., 2002; Nosyreva and Huber, 2006). This insensitivity is due to the de-repression of FMRP, which causes increased basal levels of mGluR-stimulated checkpoint proteins (Thomazeau et al., 2020). Hence in FXS, mGluR-LTD is decoupled from protein synthesis/mTOR activation. Interestingly in *Fmr1* KO mice, mTOR phosphorylation is increased in embryonic neocortex and in postnatal hippocampus samples (Sharma et al., 2010; Casingal et al., 2020).

### Mitogen-Activated Protein Kinase

The MAPK signaling pathway includes extracellular signal-Regulated Kinase 1 and 2 (ERK1 and ERK2), which are essential for neuronal transcriptional events, including synaptic plasticity, learning and memory (Thomas and Huganir, 2004). When *Mecp2* KO mice are treated with recombinant human IGF1, both AKT, ERK1, and ERK2 levels are increased in conjunction with the increase of the post-synaptic marker PSD95 (Castro et al., 2014). The MAPK pathway is upregulated in *Fmr1* KO mice, and is modulated by NNZ-2566 (Deacon et al., 2015), suggesting that mechanisms controlling plasticity may be potential targets of therapeutics in FXS and RTT, although the mechanisms of action of NNZ-2566 requires further investigation.

### PSD95

Also at the synaptic level, MeCP2 and FMRP control the expression and localization of PSD95, which is strongly related to synaptic strength in excitatory synapses. In the hippocampus, MeCP2 controls the number of glutamatergic synapses. VGLUT1 and PSD95, which are respectively pre- and postsynaptic markers, and they are both downregulated upon loss of MeCP2 in mutant mice. Conversely, a two-fold increase of MeCP2 expression, determines an increase in density and colocalization of these two markers (Chao et al., 2007). PSD95 levels are rescued by (1-3)IGF1 to levels comparable in wild type animals (Tropea et al., 2009) achieving the same phenotype as double mutants for loss and doubling of MeCP2 (Collins et al., 2004; Chao et al., 2007). PSD-95 expression is also deregulated in *Fmr1* KO mice. FMRP binds to PSD95 mRNA *in vivo* (Todd et al., 2003; Zalfa et al., 2007), suggesting that FMRP stabilizes the PSD95 transcript, leading to adequate expression levels of PSD95 (Zalfa et al., 2007).

### Bioenergetics

All energy-demanding activities, including synaptic function and plasticity, are dependent on bioenergetics, which appears to be dysfunctional in both RTT and FXS. Recent works in both FXS (Mithal and Chandel, 2020) and RTT (Neul et al., 2020) pointed at dysfunctions in mitochondria: the organelles devoted to produce the energy necessary for neuronal function. Alteration in bioenergetics has been proposed for RTT (Jin et al., 2015; Crivellari et al., 2021) and FXS (D’Antoni et al., 2019), and reflect the evolutionary need to match the cognitive function with the capacity of producing the adequate supply of energy to fulfill the requests. The link between energy metabolism and cognition has been well reviewed, and it appears clear that several brain disorders are now depending on the capacity of the organism to provide the fuel requested by the brain and to control the number of oxidative species, which are the natural side products of aerobic metabolism. Therefore, the ability to produce energy goes hand in hand with the capacity to control the reactive oxygen species and the possible damage created by an excess of these radicals. Considering the oxidative stress in FXS, it is interesting to underline that El Bekay et al. (2007) found that *Fmr1* KO mouse brains have higher levels of reactive oxygen species, nicotinamide adenine dinucleotide phosphate (NADPH)-oxidase activation, lipid peroxidation and protein oxidation compared to wild type mice. In the cortex of *Fmr1* KO it was also reported an increased level of metabolites that result from the attack of unsaturated lipids by the superoxide anion (Maurin et al., 2014). In normal conditions, the superoxide anion is detoxified by Sod1, the level of which is reduced in the absence of FMRP (Bechara et al., 2009), thus providing a source of oxidative stress (Maurin et al., 2014).

It is also worth mentioning that there is a two-way interaction between the systems controlling the production of energy in the cell, and the ion homeostasis (Castaldo et al., 2009), and that such interaction controls the onset and progression of neurodegeneration. These additional mechanisms should be taken into account for uncovering the underlying mechanisms in brain disorders and for designing to routes of treatment.

## The Contributions of Astrocytes

Considering that non-neuronal cells are involved in synaptic function and plasticity, we now discuss the contribution of astrocytes in cellular mechanisms of RTT and FXS (Table 6).

**TABLE 6:** Astrocyte function in Rett syndrome and Fragile X syndrome.

Source	Region/Sample	Experiment	Findings	References
Mecp2^–/y^	Hippocampal neurons (P1), cortical astrocytes (P1–P2)	Astrocyte/neuron co-culture, immune-cytochemistry	Mecp2^–/y^ astrocytes negatively influence dendrite arborization	Ballas et al., 2009
Mecp2^*Stop/y*^	Hippocampal neurons (3 months +)	Immunohistochemistry, behavioral assessment	Re-expression of *Mecp2* rescues dendrite morphology, locomotor and respiratory phenotypes	Lioy et al., 2011
Mecp2^*tm1*.1*Bird*^	Primary astrocyte cultures (P1) from cerebral cortex	Expression microarray, ChIP-seq	Astrocytes express a unique gene profile incl. synaptic genes	Yasui et al., 2013
*Fmr1* ^–/y^	Hippocampal neurons, Primary astrocyte cultures (P0-P1)	Astrocyte/neuron co-culture, immune-cytochemistry	*Fmr1* astrocytes-neuron co-cultures results in abnormal increased dendritic protrusions	Jacobs and Doering, 2010
*Fmr1* ^–/y^	Conditional knockout and conditional restored astrocytes (P24–P30, P38–P45), cortical slices	Behavioral assessment, Whole cell patch clamp	Loss of FMRP: ↑Cortical activity, ↑Locomotor activity, ↓Social novelty preference and memory acquisition deficits; Corrected by *Fmr1* reactivation	Jin et al., 2021
*Fmr1* ^–/y^	iPSC from patients with FXS differentiated into astrocytes	RNA sequencing, immunocytochemistry.	↑uPA expression that alters neuronal phosphorylation of TrkB	Peteri et al., 2021

*Main findings of studies reporting on astrocyte function and morphology in Rett syndrome and Fragile X syndrome. Abbreviations*: Mecp2*, methyl-CpG binding protein 2 gene; *Fmr1*, fragile X mental retardation 1 gene, FRMP, fragile X mental retardation protein; iPSC, induced pluripotent stem cell; FXS, Fragile X syndrome; uPA, urokinase plasminogen activator; TrkB tropomycin receptor kinase B.*

Astrocytes have been largely studied in RTT with a smaller body of research carried out in FXS. Astrocytes express both MeCP2 and FMRP. There is clear evidence that glial cells support normal neuronal growth and morphology, and in fact, both in RTT and FXS the co-culturing of astrocytes and neurons influence the morphology of neuronal arborization. In RTT coculturing of *Mecp2*^–/y^ astrocytes with wildtype hippocampal neurons stunts dendrite arborization and cannot sustain typical cell growth. Conversely, culturing *Mecp2* KO neurons with wildtype astrocytes results in typical dendrite morphology (Ballas et al., 2009). Re-expression of *Mecp2* in astrocytes of *Mecp2* deficient mice improves locomotor and respiratory phenotypes; moreover, re-expression of *Mecp2* in astrocytes rescues mutant neuron dendritic morphology (Lioy et al., 2011). Additionally, astrocyte gene expression profiling has identified uniquely dysregulated genes because of *Mecp2* deficiency. These genes include *Cntn1*, *Syn2*, *Gabrg1*, and *Gria1*, which function at the tripartite synapse (Yasui et al., 2013). These studies suggests that MeCP2 deficiency in astrocytes contribute to the RTT phenotype.

Fragile X mental retardation protein is expressed in astrocytes and cocultures of *Fmr1* KO astrocytes with wildtype hippocampal neurons result in neurons with abnormal increased dendritic protrusions (Jacobs and Doering, 2010). While there is an increase in dendritic density, there is reduction in overall dendrite length. There is also a significant decrease in pre- and postsynaptic proteins. Interestingly, coculturing *Fmr1* KO neurons with wildtype neurons rescues the morphological abnormalities to near wildtype phenotype (Cheng et al., 2012). These results suggest that astrocytes are implicated in neuronal dendritic morphology also in FXS.

Recently, Jin and co-workers (Kang et al., 2021) provided evidence that FMRP mediates synaptic connectivity through astrocytes and therefore controls learning and behavior. By using conditional knockout (cKO) and conditional restored (cON) mice in astrocytes, they find that loss of FMRP results in cortical hyperactivity, increased locomotor activity, reduced social novelty preference and deficit of memory acquisition. Reactivation of the astrocyte *Fmr1* rescues these phenotypes (Jin et al., 2021).

Interestingly, in human FXS astrocytes generated from human induced pluripotent stem cells an increased expression of urokinase plasminogen activator (uPA), which modulates degradation of extracellular matrix, was reported. Increased uPA augmented neuronal phosphorylation of TrkB within the docking site for the phospholipase-Cγ1 (PLCγ1), indicating effects of uPA on neuronal plasticity (Peteri et al., 2021) and connecting this molecular alteration to the BDNF pathway.

## Conclusion

In recent years it has become clear that neurodevelopmental disorders share common molecular mechanisms, and that their complex clinical presentation results from the interaction of genetic and environmental factors. Genetic studies are growing in power and are showing that genes involved in synaptic function are major risk factors for neurodevelopmental disorders, but other mechanisms, such as neuroimmunity, and mitochondrial functions are also emerging as contributors to the onset and progression of several disorders. In this context, the study of convergent and divergent mechanisms between RTT and FXS can be instructive in understanding the general biological mechanisms that underlie a variety of disorders, including those with multi-genic components. Encouraging results in therapeutic strategies for RTT (Glaze et al., 2019) and FXS (Berry-Kravis et al., 2020) confirm the perspective that some treatments can be effective for multiple conditions, and foster research that uncover overlapping mechanisms across disorders. In line with this perspective, our review suggests that the analysis of common and divergent mechanisms controlling synaptic function and plasticity can instruct new criteria for the classification of neurodevelopmental disorders, with applications to diagnosis, prognosis, and drug discovery.

## Author Contributions

DT, SS, and SB contributed to the initial draft of the manuscript. DT, BB, and SB revised the manuscript. All authors discussed and provided the input on the manuscript.

## Conflict of Interest

The authors declare that the research was conducted in the absence of any commercial or financial relationships that could be construed as a potential conflict of interest.

## Publisher’s Note

All claims expressed in this article are solely those of the authors and do not necessarily represent those of their affiliated organizations, or those of the publisher, the editors and the reviewers. Any product that may be evaluated in this article, or claim that may be made by its manufacturer, is not guaranteed or endorsed by the publisher.
